# Supplementary material for: Identification of key genes, pathways and potential therapeutic agents for liver fibrosis using an integrated bioinformatics analysis
Source: PeerJ. 2019 Mar 22;7:e6645. doi: 10.7717/peerj.6645 (PMC6432904; doi:10.7717/peerj.6645)
Supplement: Data S1 [file peerj-07-6645-s001.docx]

**Supplementary data1**. Primers of 25 hub genes

| Genes | Aceesssion no. | Forward Primer | Reverse Primer |
| --- | --- | --- | --- |
| COL1A2 | NM_000089 | 5'-GGCCCTCAAGGTTTCCAAGG-3' | 5'-CACCCTGTGGTCCAACAACTC-3' |
| COL1A1 | NM_000088 | 5'-GAGGGCCAAGACGAAGACATC-3' | 5'-CAGATCACGTCATCGCACAAC-3' |
| COL6A3 | NM_057166 | 5'-CATAACCGCTGTGCGGAAAAT-3' | 5'-TCATCTAGGGACTTACCACCTG-3' |
| COL3A1 | NM_000090 | 5'-TTGAAGGAGGATGTTCCCATCT-3' | 5'-ACAGACACATATTTGGCATGGTT-3' |
| COL5A2 | NM_000393 | 5'-GACTGTGCCGACCCTGTAAC-3' | 5'-CCTGGACGACCACGTATGC-3' |
| COL5A1 | NM_000093 | 5'-GCCCGGATGTCGCTTACAG-3' | 5'-AAATGCAGACGCAGGGTACAG-3' |
| COL4A1 | NM_001845 | 5'-GTGTTGACGGCTTACCTGGAGAC-3' | 5'-CGGGAAGACCTGGCAAACCTTT-3' |
| COL4A2 | NM_001846 | 5'-TTATGCACTGCCTAAAGAGGAGC-3' | 5'-CCCTTAACTCCGTAGAAACCAAG-3' |
| COL4A3 | NM_000091 | 5'-GCAGTGGTTCTAAGGGTGAGC-3' | 5'-GAAAGCCAAAGAATCCCGGAG-3' |
| COL4A4 | NM_000092 | 5'-TCGGTGGCTTCCTCCTGGTT-3' | 5'-ATCTGTCGTTTCTCTGGGCATAGTG-3' |
| DCN | NM_133506 | 5'-ATGAAGGCCACTATCATCCTCC-3' | 5'-GTCGCGGTCATCAGGAACTT-3' |
| COL14A1 | NM_021110 | 5'-CACTTCCTACACGACCACCAACTT-3' | 5'-GGTTCCATCTGTGCCAATCTTGTTC-3' |
| LUM | NM_002345 | 5'-TAACTGCCCTGAAAGCTACCC-3' | 5'-GGAGGCACCATTGGTACACTT-3' |
| COL15A1 | NM_001855 | 5'-CTGCAGAAAGCGCATTTGGT-3' | 5'-GTGGAGGCAGAAGCTGATGT-3' |
| THBS2 | NM_003247 | 5'-ATAGACAGCTTCGCTCTGGAC-3' | 5'-CAAACCCCTGAAGTGACTCTC-3' |
| FBN1 | NM_000138 | 5'-TTTAGCGTCCTACACGAGCC-3' | 5'-CCATCCAGGGCAACAGTAAGC-3' |
| ITGB8 | NM_002214 | 5'-ACCAGGAGAAGTGTCTATCCAG-3' | 5'-CCAAGACGAAAGTCACGGGA-3' |
| CDH11 | NM_001797 | 5'-AGAGGTCCAATGTGGGAACG-3' | 5'-GGTTGTCCTTCGAGGATACTGT-3' |
| ADAMTS2 | NM_021599 | 5'-GACACGGGCCACGATGAATA-3' | 5'-GGTGACAGGAGCATAGCCTT-3' |
| CTGF | NM_001901 | 5'-AAAAGTGCATCCGTACTCCCA-3' | 5'-CCGTCGGTACATACTCCACAG-3' |
| VCAN | NM_001126336 | 5'-GTAACCCATGCGCTACATAAAGT-3' | 5'-GGCAAAGTAGGCATCGTTGAAA-3' |
| PCOLCE2 | NM_013363 | 5'-TGCCGCTATGACTTTGTGGAT-3' | 5'-GGCATCAGAAATCATCTGCACC-3' |
| SPP1 | NM_001251830 | 5'-GAAGTTTCGCAGACCTGACAT-3' | 5'-GTATGCACCATTCAACTCCTCG-3' |
| VWF | NM_000552 | 5'-CCGATGCAGCCTTTTCGGA-3' | 5'-TCCCCAAGATACACGGAGAGG-3' |
| CTSK | NM_000396 | 5'-ACTCAAAGTACCCCTGTCTCAT-3' | 5'-CCACAGAGCTAAAAGCCCAAC-3' |

The primers were downloaded from Primerbank or designed with Primer6 and verified by Oligo7.
